# Ovariectomy and Subsequent Treatment with Estrogen Receptor Agonists Tune the Innate Immune System of the Hippocampus in Middle-Aged Female Rats

**DOI:** 10.1371/journal.pone.0088540

**Published:** 2014-02-13

**Authors:** Miklós Sárvári, Imre Kalló, Erik Hrabovszky, Norbert Solymosi, Zsolt Liposits

**Affiliations:** 1 Laboratory of Endocrine Neurobiology, Institute of Experimental Medicine, Hungarian Academy of Sciences, Budapest, Hungary; 2 Faculty of Information Technology, Pázmány Péter Catholic University, Budapest, Hungary; 3 Department of the Physics of Complex Systems, Eötvös Loránd University, Budapest, Hungary; Hosptial Infantil Universitario Niño Jesús, CIBEROBN, Spain

## Abstract

The innate immune system including microglia has a major contribution to maintenance of the physiological functions of the hippocampus by permanent monitoring of the neural milieu and elimination of tissue-damaging threats. The hippocampus is vulnerable to age-related changes ranging from gene expression to network connectivity. The risk of hippocampal deterioration increases with the decline of gonadal hormone supply. To explore the impact of hormone milieu on the function of the innate immune system in middle-aged female rats, we compared mRNA expression in the hippocampus after gonadal hormone withdrawal, with or without subsequent estrogen replacement using estradiol and isotype-selective estrogen receptor (ER) agonists. Targeted profiling assessed the status of the innate immune system (macrophage-associated receptors, complement, inhibitory neuronal ligands), local estradiol synthesis (P450 aromatase) and estrogen reception (ER). Results established upregulation of macrophage-associated (Cd45, Iba1, Cd68, Cd11b, Cd18, Fcgr1a, Fcgr2b) and complement (C3, factor B, properdin) genes in response to ovariectomy. Ovariectomy upregulated Cd22 and downregulated semaphorin3A (Sema3a) expression, indicating altered neuronal regulation of microglia. Ovariectomy also led to downregulation of aromatase and upregulation of ERα gene. Of note, analogous changes were observed in the hippocampus of postmenopausal women. In ovariectomized rats, estradiol replacement attenuated Iba1, Cd11b, Fcgr1a, C3, increased mannose receptor Mrc1, Cd163 and reversed Sema3a expression. In contrast, reduced expression of aromatase was not reversed by estradiol. While the effects of ERα agonist closely resembled those of estradiol, ERβ agonist was also capable of attenuating the expression of several macrophage-associated and complement genes. These data together indicate that the innate immune system of the aging hippocampus is highly responsive to the gonadal hormone milieu. In ovariectomized female rats, estradiol replacement exerts potent immunomodulatory effects including attenuation of microglia sensitization, initiation of M2-like activation and modulation of complement expression by targeting hippocampal neurons and glial cells through ERα and ERβ.

## Introduction

The hippocampus plays a crucial role in learning and memory, and it is essential for planning and creativity [1]. This brain area is vulnerable to age-related changes ranging from gene expression to neuronal network connectivity [2], [3]. Ongoing neurogenesis, which helps to maintain normal hippocampal function, is impaired dramatically during aging [4]. Age-related alterations are implicated in the development of neurodegenerative diseases [5]. Changes in gene expression start at midlife [6], which overlaps with the time of cessation of estrogen and progesterone synthesis by the ovaries [7]. In rodents, alterations of the hippocampal transcriptome also begin at midlife [8]. Estrogen and progesterone receptors are expressed in the hippocampus [9]–[12]. ERα and ERβ are of particular interest as they mediate the effects of 17β-estradiol (E2) on gene expression [13], [14], intracellular signaling [15], [16] and synaptic plasticity [17], [18]. Besides synaptic plasticity, E2 modulates glial functions and regulates the immune response [19]–[23]. By the time of menopause, declining gonadal hormone supply is associated with increased risks of hippocampal deterioration and memory disturbances [24], [25].

Microglial cells, which form part of the innate immune system, play an important role in the maintenance of hippocampal functions via constant monitoring of the neural milieu to eliminate tissue-damaging threats. In addition, surveying microglia acutely modulate synaptic activity by regulating the number of functional synapses in organotypic slices [26]. Microglia also remodel neuronal circuits in the postnatal retinogeniculate system in an activity and complement-dependent manner [27]. Microglial cells express steroid hormone receptors [28]. While there is consensus about the expression of ERα, expression of ERβ is rather controversial [28]–[31]. Independent studies have detected only ERα at mRNA and protein level in microglia isolated from adult mice [28], [30]. Anti-inflammatory effects of E2 are mediated primarily through ERα [19], but a recent study has demonstrated that the androgen metabolite 5-androsten-3β,17β-diol can suppress inflammatory processes of microglia and astrocytes via acting through ERβ [29]. Another laboratory has provided immunohistochemical data that support ERβ expression in mouse microglia [31]. Progesterone and its metabolite allopregnanolone modify the action of E2 and exert immunomodulatory effects [32]. Microglia continuously survey the local environment via receptors for neurotransmitters, danger signals, chemokines, cytokines and complement activation fragments among others [33]. Receptors for danger signals and inhibitory neuronal ligands represent the accelerator and the break for the transition from surveying to effector microglia, respectively [33].

In rat models of normal aging, molecular and functional alterations of microglia indicate the elevation of microglia reactivity. Microglial expression of MHCII [34], [35] and other cell surface markers such as CD11b [36], CD68 [37], CD80, CD86 [38] increases in the normal aged brain. The initial shift in microglia phenotype may occur at midlife [6] coinciding with the menopausal transition. Therefore, we hypothesize that menopause upsets the delicate balance between activation signals and inhibitory neuronal ligand control resulting in a shift of the microglia phenotype in the aging female hippocampus.

Middle-aged ovariectomized rats are widely used to study behavioral [39], [40], cellular [41] and molecular [17], [42] changes related to menopause. Here, we studied the hippocampal transcriptome of this animal model in order to explore the effects of ovariectomy and subsequent estrogen replacement; transcripts analyzed were related to the innate immune system including microglia, complement (especially components of the alternative pathway) and cytokines. In replacement studies we used E2 and isotype-selective ER agonists. As the expression of macrophage-associated genes is low in surveying microglia at this age [35], we followed mRNA expression, which allowed us to quantitatively typify gene expression. Results of the present study provide evidence that ovariectomy of middle-aged rats increases the expression of macrophage-associated receptors, components of the alternative activation pathway of complement and inhibitory neuronal ligands for microglia. We show that estrogen replacement attenuates the shift in the microglia phenotype, partly restores neuronal control of microglia and modulates complement expression. These data suggest that ovariectomy-dependent changes in the innate immune system can be attenuated by timely estrogen replacement.

## Materials and Methods

### Ethics Statement

All studies were carried out with permission from the Animal Welfare Committee of the Institute of Experimental Medicine, Hungarian Academy of Sciences (Permission Number: A5769-01) and in accordance with the legal requirements of the European Community (Decree 86/609/EEC). Animal experimentation described was conducted in accord with accepted standards of animal care.

### Reagents

E2 and diarylpropionitrile (DPN) were obtained from Sigma (St.Louis, MO, USA) and Tocris (Ellisville, MO, USA), respectively. 3,17β-dihydroxy-19-nor-17α-pregna-1,3,5(10)-triene-21,16α-lactone (LE2) was synthesized and kindly provided for this study by Gedeon Richter Plc [43]. Alzet osmotic minipumps (model 2004) were purchased from Durect (Cupertino, CA, USA). Reverse transcription reagents, microfluidic cards and PCR master mixes were ordered from Applied Biosystems (Foster City, CA, USA).

### Treatments of Experimental Animals

Female Harlan-Wistar rats were purchased from Toxicoop (Budapest, Hungary) and were housed under standard laboratory conditions with unrestricted access to phytoestrogen-free rodent diet (Harlan Teklad Global Diets, Madison, WI, USA). The following experimental groups were applied: middle-aged (13 month old) female rats (M group), middle-aged ovariectomized (OVX) rats (M/OVX group) and M/OVX rats with estrogen replacement. Replacement was performed with E2 (M/OVX+E2 group), ERα agonist LE2 (M/OVX+LE2 group) and ERβ agonist DPN (M/OVX+DPN group). Middle-aged (n = 26) rats underwent bilateral ovariectomy except for the sham-operated M group (n = 8). All rats were housed individually after surgery, and ten days later received treatments with vehicle or ER agonists. Treatments in middle-aged OVX rats were carried out as described earlier [43]. In brief, Alzet 2004 osmotic minipumps filled either with E2 (0,33 mg/ml in propylene-glycol, n = 8), LE2 (3,33 mg/ml in propylene-glycol, n = 5), DPN (3,33 mg/ml in propylene-glycol, n = 5), or the vehicle only (n = 8, M/OVX group) were implanted subcutaneously for 29 days. Concentrations of E2 and isotype-selective ER agonists were calculated to produce a release rate of 2.0µg/d and 20 µg/d, respectively. Treatments with E2 and LE2 at these doses result in a similar increase in uterine weight, while DPN causes no change [43]. Body weights were measured to test efficacy of the treatments.

### Sample Preparation

On the day of sample preparation, animals were deeply anesthetized and perfused transcardially with 100 ml of cold fixative solution containing 10% RNA*later* (Qiagen, Heidelberg, Germany) in phosphate buffered saline. In all experiments, the same procedure was followed for the preparation of the hippocampal formation.

### Quantitative Real-time PCR

The hippocampi were prepared and total RNA was isolated using the RNeasy Lipid Tissue Kit (Qiagen, Hilden, Germany). RNA analytics included A260 nm/A280 nm readings using a Nanodrop Spectrophotometer and capillary electrophoresis using Nano RNA Chips on 2100 Bioanalyzer (Agilent, Santa Clara, CA, USA). All RNA samples displayed high RNA integrity numbers (RIN >8.2).

Custom TaqMan microfluidic cards were designed to study in depth the regulation of ninety-six genes by quantitative real-time PCR. Microfluidic cards (Applied Biosystems, Foster City, CA, USA) were preloaded by the manufacturer with selected inventoried assays for genes of our interest (Table 1). We used glyceraldehyde-3-phosphate dehydrogenase and hypoxanthine guanine phosphoribosyl-transferase as housekeeping genes. Expression of these genes did not vary among treatment groups of the study. Each assay consisted of a FAM dye-labeled TaqMan MGB probe and two PCR primers. Reverse transcription and real-time PCR were run as described earlier [43]. The ViiA7 RUO (Applied Biosystems) software and relative quantification against calibrator samples (ΔΔCt) were used for data analysis. A computed internal control corresponding to the geometric mean of cycle threshold (Ct) values of the selected housekeeping genes was used for ΔCt calculation [44]. The use of TaqMan chemistry allowed comparison of ΔCt values which correlated with transcript levels. Based on ΔCt values, macrophage-associated genes were ranked into three categories based on their abundant (0<ΔCt<1), moderate (1<ΔCt<5) or low (ΔCt>5) level of mRNA expression. Relative quantity (RQ = 2^−ΔΔCt^) was used to characterize gene expression in the various experimental groups. PCR experiments conformed to minimum information for publication of quantitative real-time PCR experiments (MIQE) guidelines [45].

**Table 1:** Expression of macrophage-associated genes and inhibitory neuronal ligands in the hippocampus of middle-aged female rats.

	TaqMan ID	ΔCt
**MACROPHAGE-ASSOCIATED GENES**		
*markers*		
Iba1	Rn00574125_g1	4.982
Cd68	Rn01495634_g1	8.305
Cd80	Rn00709368_m1	10.30
Cd86	Rn00571654_m1	7.886
RT1-EC2	Rn03034964_u1	8.432
Cd74	Rn00565062_m1	3.941
Mrc1	Rn01487342_m1	8.548
Cd163	Rn01492519_m1	9.177
*phagocytic receptors*		
Cd11b	Rn00709342_m1	5.333
Cd18	Rn01427948_m1	6.386
Fcgr1a	Rn01762682_m1	8.407
Fcgr2b	Rn00598391_m1	6.818
Fcgr2a	Rn00821543_g1	4.860
*recognition receptors*		
Cd14	Rn00572656_g1	9.929
Tlr3	Rn01488472_g1	9.142
Tlr4	Rn00569848_m1	7.975
Tlr9	Rn01640054_m1	8.768
*receptors for inhibitory neuronal ligands*		
Cd45	Rn00709901_m1	7.350
Nrp1	Rn00686106_m1	1.981
Sirpa	Rn00564609_m1	0.845
Cd200r1	Rn00576646_m1	11.53
Cx3cr1	Rn02134446_s1	3.657
**NEURONAL GENES**		
*inhibitory ligands for microglia*		
Cd22	Rn01457837_m1	10.54
Sema3a	Rn00436469_m1	7.487
Cd47	Rn01763248_m1	4.125
Cd200	Rn01646320_m1	3.268
Cx3cl1	Rn00593186_m1	0,168

mRNA expression was studied by real-time PCR using TaqMan chemistry. ΔCt represents the difference between the Ct of a given gene and of the endogenous control (geometric mean of the Ct values of the selected housekeeping genes glyceraldehyde-3-phosphate dehydrogenase and hypoxanthine guanine phosphoribosyl-transferase, 21.887±0.296). Ct, cycle threshold.

### Analysis of Human Microarray Data

The files of data set GSE11882 [6], deposited in Gene Expression Omnibus, contained microarray data from the hippocampus. Sample size (n), average age (age) in years and standard deviation of ages (SD) were used to characterize the premenopausal (n_HC_ = 9, age_HC_ = 39.4, SD_HC_ = 8.0) and postmenopausal (n_HC_ = 5, age_HC_ = 71.2, SD_HC_ = 4.4) hippocampus data. Pre- and postmenopausal groups consisted of nine and five individuals, respectively. Only changes with fold change (FC)>1.5 (upregulation) and FC<0.66 (downregulation) were considered as FC is the most reliable parameter in the case of low sample numbers [46]. Raw microarray data were pre-processed for analysis by GC robust multi-array average (GCRMA) [47]. From the expression set, probesets were selected based on the relevance to rat data.

### Statistical Analysis

In PCR data evaluation, group data were expressed as RQ(mean)±standard deviation (SD). Statistical significance of the changes in gene expression was analyzed using ANOVA followed by Newman-Keuls post-hoc test (Statistica software version 11.0, StatSoft Inc., Tulsa, OK). Homocedasticity and normality satisfied the criteria for running ANOVA. In correlation analysis Pearson’s coefficient was estimated.

In microarray data evaluation, difference analysis of gene expression was performed by linear models combined with Bayesian methods [48], p was adjusted by the false discovery rate-based method [49]. In statistical and data mining work, Bioconductor packages [50] in R-environment were used.

## Results

### Expression of Immune-related Genes in the Hippocampus of Middle-aged Female Rats

We investigated mRNA expression of macrophage-associated genes (Table 1) in the hippocampus. Under physiological conditions, these genes are expressed predominantly in microglial cells, although in the case of toll-like receptors astroglial expression has also been reported [51]. Among macrophage-associated genes, we measured moderate to low mRNA level of microglia response factor Iba1 and Fcgr2a, the rest of the genes tested had low mRNA levels indicating a very weak macrophage character of microglia in the hippocampus of middle-aged female rats. On the other hand, microglial receptors for inhibitory neuronal ligands showed significant expression. We detected high mRNA level of signal-regulatory protein α (Sirpa), moderate levels of neuropilin 1 (Nrp1), fractalkine receptor (Cx3cr1), low levels of leukocyte common antigen (Cd45) and very low levels of Cd200r (Table 1).

We also investigated the expression of complement and proinflammatory cytokine genes (Table 2). Both neurons and glial cells express complement components and their regulators. We found moderate mRNA levels of C3, Cfh, Crry and Cd59, other complement genes had low expression levels. We measured very low levels of proinflammatory cytokine mRNAs (Table 2).

**Table 2:** Expression of complement and cytokine genes in the hippocampus of middle-aged female rats.

	TaqMan ID	ΔCt
**COMPLEMENT AND CYTOKINE GENES**		
*complement and complement regulators*		
C1qa	Rn01519903_m1	6.625
Serping1	Rn01485600_m1	6.023
C3	Rn00566466_m1	4.864
Cfb	Rn01526084_g1	6.618
Cfd	Rn01535436_g1	8.955
Cfh	Rn00590326_m1	4.580
Cfp	Rn01430864_m1	9.820
Crry	Rn00570775_m1	4.068
Cd55	Rn00709472_m1	7.447
Cd59	Rn00563929_m1	2.963
*proinflammatory cytokines*		
Tnf	Rn01525859_g1	10.141
Il1b	Rn00580432_m1	13.147
Il6	Rn01410330_m1	11.088
Il12b	Rn00575112_m1	18.053

mRNA expression of complement and proinflammatory cytokine genes was measured by real-time PCR using TaqMan assays. ΔCt represents the difference between Ct of a given gene and of the endogenous control. Ct, cycle threshold.

### Impact of Ovarian Hormone Depletion and Subsequent Estrogen Treatment on the Expression of Macrophage-associated Genes

We examined the effect of ovarian hormones on mRNA expression of macrophage-associated genes that encode microglial markers, phagocytic receptors, recognition molecules and receptors for inhibitory neuronal ligands.

#### Regulation of microglial marker genes

**Figure 1:**
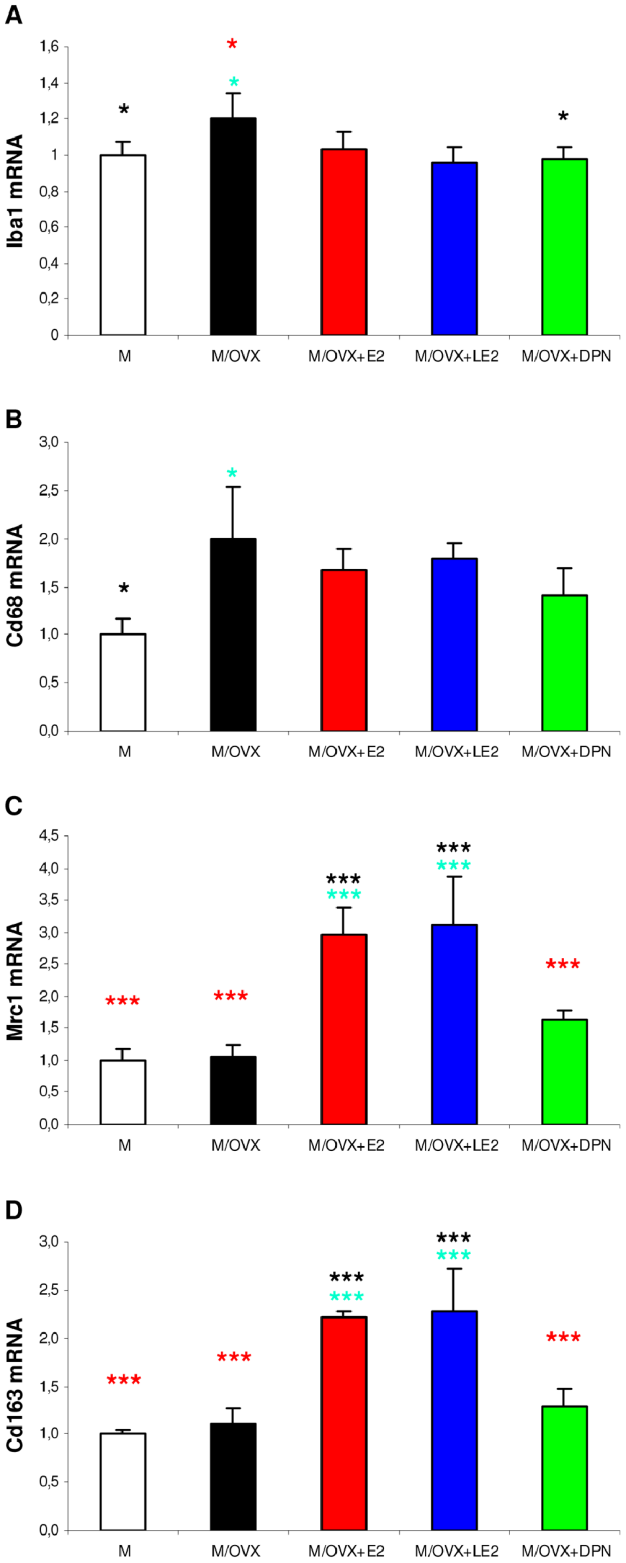
The effect of ovariectomy and treatment with E2 and isotype-selective ER agonists on mRNA expression of microglia marker genes. Expression of microglia marker Iba1 (A), Cd68 (B), and M2 marker Mrc1 (C) and Cd163 (D) in the hippocampus of middle-aged (M), middle-aged ovariectomized (M/OVX) and M/OVX rats treated with E2 (M/OVX+E2), LE2 (M/OVX+LE2) and DPN (M/OVX+DPN) was determined by real-time PCR. Error bars show SD of five samples for each group. ANOVA identified statistically significant treatment effects for each gene (p = 0.009 for Iba1, p = 0.014 for Cd68, p<0.001 for M2 markers). Turquoise, black and red asterisks mark statistically significant group differences compared to M, M/OVX and M/OVX+E2 animals, respectively, by Newman-Keuls post hoc test. * corresponds to 0.01<P<0.05 and *** to P<0.001. M, middle-aged female rat; M/OVX, middle-aged OVX rat; M/OVX+E2, M/OVX rats treated with E2; M/OVX+LE2, treated with 16α-LE2; M/OVX+DPN, treated with DPN. P, p value.

In middle-aged OVX rats, expression of Iba1 (Fig. 1A) and Cd68 (Fig. 1B) increased 1.2-fold (p = 0.036) and 1.8-fold (p = 0.027), respectively, compared to middle-aged controls. Cd80 showed a similar increase as Iba1 (RQ = 1.367, p = 0.052), whereas Mrc1 (Fig. 1C), Cd163 (Fig. 1D) and Cd86 (data not shown) did not change after ovariectomy. Subsequent E2 replacement attenuated ovariectomy-evoked upregulation of Iba1 (Fig. 1A) and Cd68 (Fig. 1B). In addition, E2 increased mRNA expression of Mrc1 (Fig. 1C) and Cd163 (Fig. 1D) compared to middle-aged control rats. In order to assess the contribution of the two ER isotypes to E2 responses, chronic treatments were carried out with either the ERα agonist LE2 [52] or the ERβ agonist DPN [53]. Both LE2 and DPN mitigated ovariectomy-induced upregulation of Iba1 and Cd68. Only inhibition of Iba1 expression by DPN reached statistical significance compared to M/OVX animals. Similar to E2, LE2 enhanced the expression of Mrc1 and Cd163 compared to M and M/OVX animals (Fig. 1).

We also explored hormonal regulation of two MHC genes, RT1-EC2 and Cd74. Ovariectomy evoked 2.1-fold increase in mRNA expression of RT1-EC2 (p = 0.031) compared to middle-aged controls. Estrogen treatments did not attenuate the alteration. Cd74 showed no hormonal regulation in the hippocampus.

#### Regulation of phagocytic receptor genes

**Figure 2:**
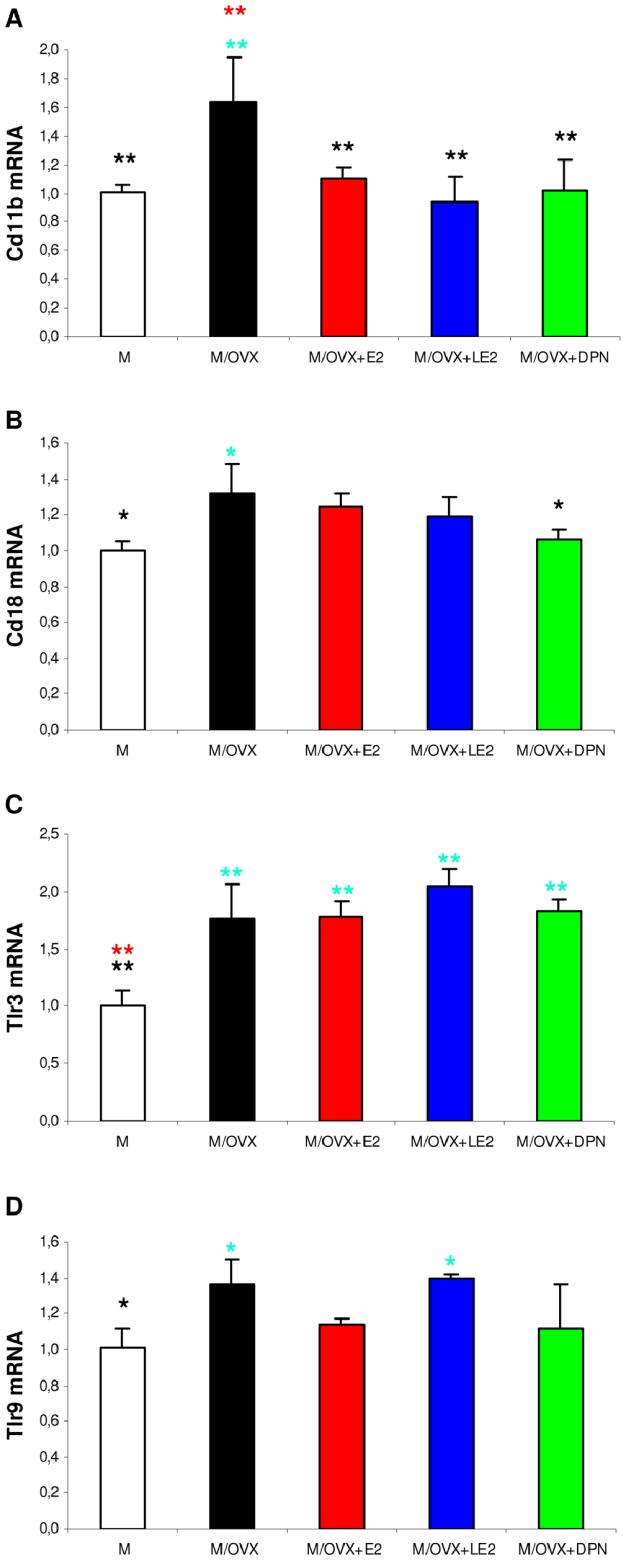
Ovariectomy-evoked changes in the expression of phagocytic and recognition receptors were responsive to ER agonists. mRNA expression of Cd11b (A), Cd18 (B), Tlr3 (C) and Tlr9 (D) in the hippocampus was determined by real-time PCR. Error bars show SD of five samples for each group. ANOVA identified statistically significant differences in gene expression among treatment groups for all genes (p<0.001 for Cd11b, p = 0.012 for Cd18, p<0.001 for Tlr3, p = 0.009 for Tlr9). Turquise, black and red asterisks mark statistically significant group differences compared to M, M/OVX and M/OVX+E2 animals, respectively, by Newman-Keuls post hoc test. * corresponds to 0.01<P<0.05 and ** to 0.001<P<0.01.

All phagocytic receptor genes except low affinity FcγR (Fcgr2b) showed moderate, but significant upregulation in middle-aged OVX rats compared to controls. For example, complement receptor 3 subunits Cd11b (Fig. 2A) and Cd18 (Fig. 2B) mRNA expression increased 1.6-fold (p = 0.008) and 1.2-fold (p = 0.025), respectively. Expression of FcγRs including Fcgr1a (RQ = 1.413, p = 0.046) and Fcgr2b (RQ = 1.437, p = 0.011) also increased after ovariectomy. E2 replacement attenuated ovariectomy-induced upregulation of Cd11b (Fig. 2A), Cd18 (Fig. 2B) and Fcgr1a (data not shown) compared to middle-aged controls. Alterations reached statistical significance compared to M/OVX animals. Isotype-selective ER agonists also reduced the expression of Cd11b and Cd18. In turn, neither E2 nor the ER agonists attenuated the expression of Fcgr2b.

#### Regulation of recognition receptors

Among recognition molecules, we found upregulation of Tlr3 (RQ = 1.755, p = 0.003) (Fig. 2C) and Tlr9 (RQ = 1.369, p = 0.042) (Fig. 2D) in ovariectomized rats compared to controls. Chronic treatment with E2 decreased Tlr9 expression (Fig. 2D), but did not alter Tlr3. Only DPN was effective to reduce mRNA expression of Tlr9 (Fig. 2D).

#### Regulation of regulatory receptors

**Figure 3:**
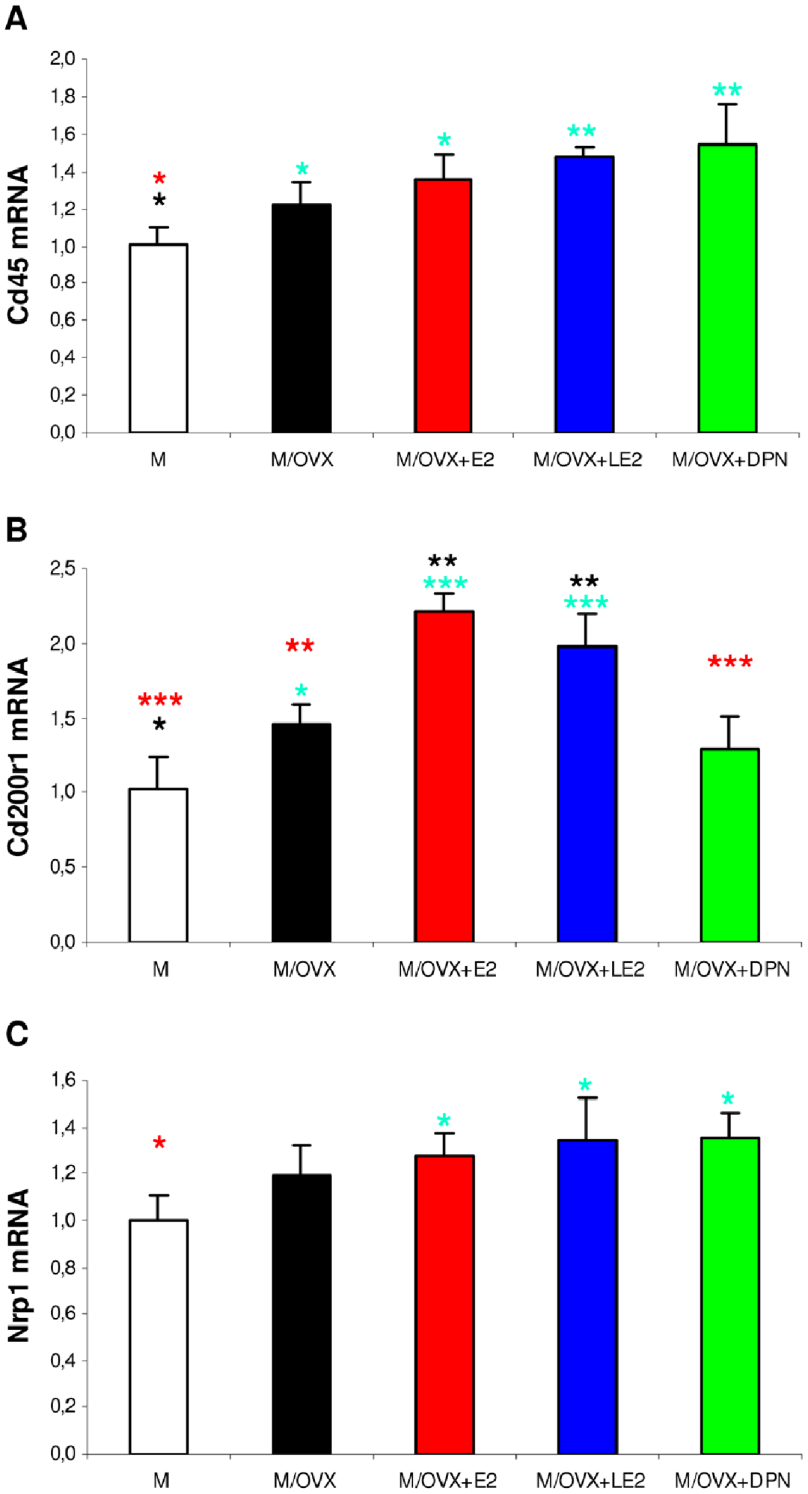
Ovarian hormone depletion and treatment with ER agonists induced changes in mRNA expression of microglial receptors for inhibitory neuronal ligands. Expression of Cd45 (A), Cd200r1 (B) and Nrp1 (C) in the hippocampus was measured by real-time PCR. Error bars show SD of five samples for each group. ANOVA revealed statistically significant treatment effects for each gene (p = 0.002 for Cd45, p = 0.014 for Nrp1, p<0.001 for Cd200r1). Turquoise, black and red asterisks mark statistically significant group differences compared to M, M/OVX, M/OVX+E2 animals, respectively, by Newman-Keuls post hoc test. * corresponds to 0.01<P<0.05, ** to 0.001<P<0.01 and *** to P<0.001.

Among microglial receptors for inhibitory neuronal ligands, mRNA expression Cd45 (Fig. 3A) and Cd200r (Fig. 3B) increased 1.2-fold (p = 0.049) and 1.5-fold (p = 0.037), respectively, in ovariectomized rats compared to controls. Nrp1 (Fig. 3C), Sirpa and Cx3cr1 expression did not alter. E2 replacement and chronic treatments with isotype-selective ER agonists increased mRNA expression of regulatory receptors (Fig. 3). Of note, E2 and LE2 significantly enhanced expression of Cd200r compared to M/OVX rats (Fig. 3B).

**Figure 4:**
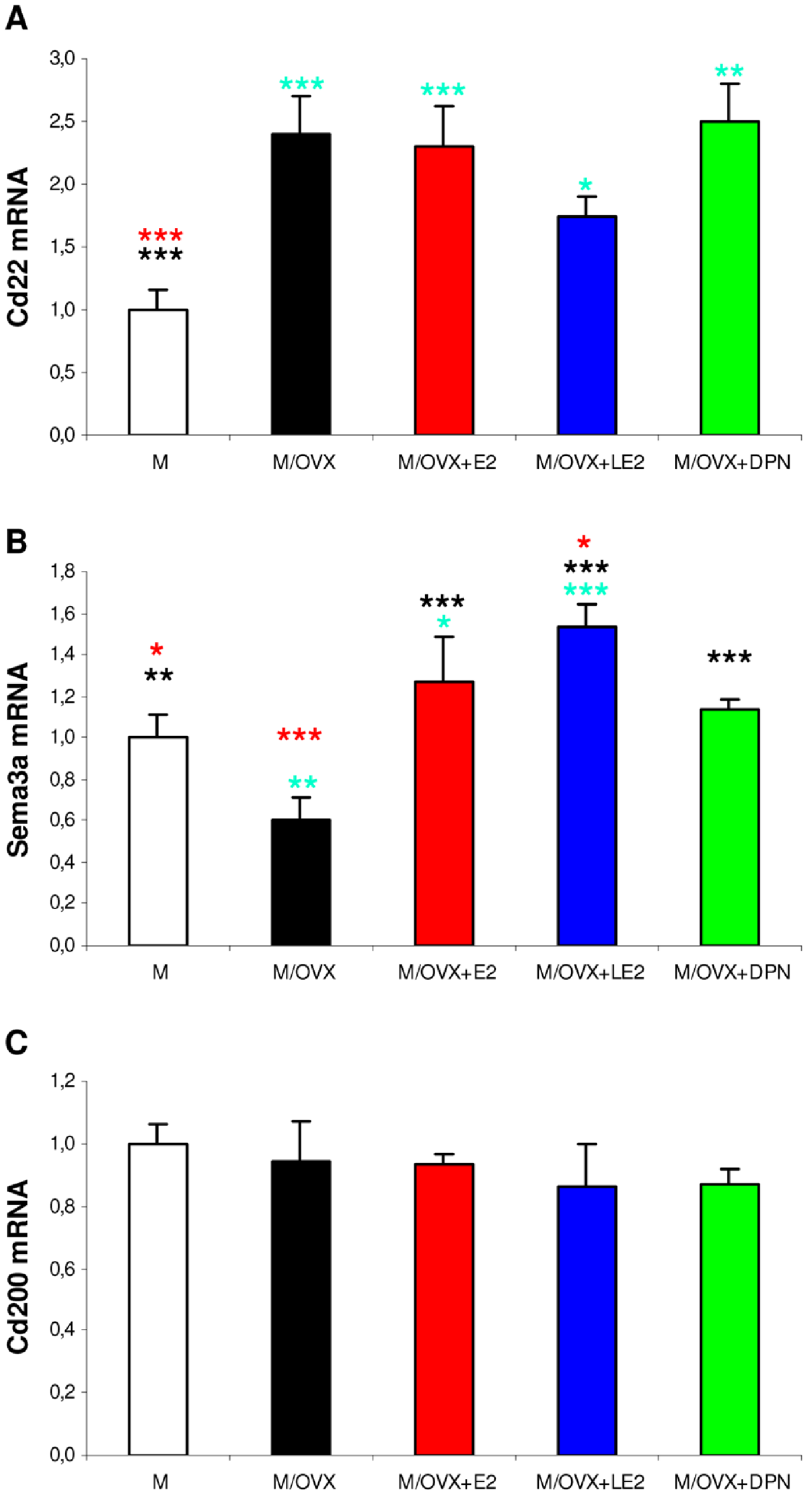
Ovariectomy and subsequent ER agonist treatments modulated mRNA expression of inhibitory neuronal ligand genes. Expression of Cd22 (A), Sema3a (B) and Cd200 (C) in the hippocampus was measured by real-time PCR. Error bars show SD of five samples for each group. There were statistically significant treatment effects in the case of Cd22 and Sema3a (p value of the ANOVA was smaller than 0.001 in both cases, p = 0.328 for Cd200). Turquoise, black and red asterisks indicate significant group differences compared to M, M/OVX, M/OVX+E2 animals, respectively, by Newman-Keuls post hoc test. * corresponds to 0.01<P<0.05, ** to 0.001<P<0.01 and *** to P<0.001.

Ovariectomy modulated expression of genes encoding inhibitory neuronal ligands for microglia as well. Expression of Cd22 enhanced 2.5-fold (p<0.001) (Fig. 4A), of Sema3a decreased 1.6-fold (p = 0.001) (Fig. 4B). Other inhibitory neuronal ligands including Cd47, Cd200 (Fig. 4C), Cx3cl1 did not change after ovariectomy. Only Sema3a expression was reversed by treatments with E2 and ERα agonist LE2 (Fig. 4B).

In addition, we investigated the expression of four astroglial markers including glial fibrillary acidic protein, colony stimulating factor 1, and glial high affinity glutamate transporters (Slc1a2, Slc1a3). These genes showed negligible alterations (data not shown).

### Effect of Ovarian Hormones and Isotype-selective ER Agonists on the Expression of Complement Genes

**Figure 5:**
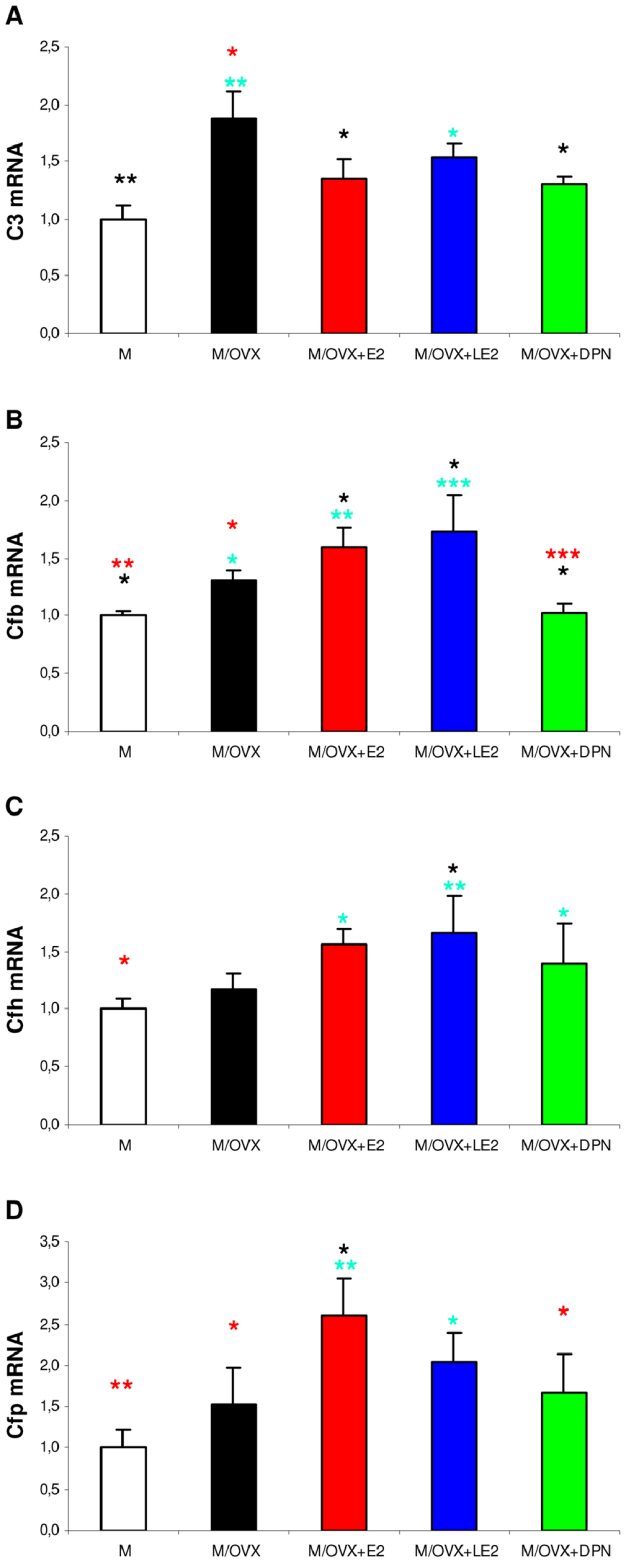
Ovariectomy and ER agonists modified the expression of complement genes. Expression of C3 (A), Cfb (B), Cfh (C) and Cfp (D) in the hippocampus of M, M/OVX, M/OVX+E2, M/OVX+LE2 and M/OVX+DPN rats was quantified by real-time PCR. Error bars show SD of five samples for each group. ANOVA identified statistically significant treatment effects for each gene (p<0.001 for C3 and Cfb, p = 0.002 for Cfh and Cfp). Turquoise, black and red asterisks mark significant group differences compared to M, M/OVX and M/OVX+E2 animals, respectively, by Newman-Keuls post hoc test. * corresponds to 0.01<P<0.05, ** to 0.001<P<0.01 and *** to P<0.001.

Neurons and glial cells express complement components and their regulators [54]. The classical and alternative complement activation pathways converge at C3 activation resulting in the release of C3a and generation of iC3b on the surface of cells rendered to phagocytosis [55]. Expression of C3, the central component of complement increased 1.9-fold (p = 0.001) in ovariectomized rats compared to controls (Fig. 5A). Factor B (Cfb), subunit of the alternative pathway C3 convertase C3bBb, showed similar but smaller (1.3-fold, p = 0.037) increase in mRNA expression than C3 (Fig. 5B). Factor D (Cfd) showed no alteration following ovariectomy. Negative regulators of C3bBb such as factor H (Cfh) (Fig. 5C), complement regulatory protein (Crry) and Cd55 did not change. Expression of positive regulator of C3bBb, properdin (Cfp) increased 1.5-fold (Fig. 5D), but the change did not reach statistical significance (p = 0.116). Cd59, an inhibitor of homologous complement lysis showed unaltered expression.

Chronic treatments with E2 and DPN reversed upregulation of C3, while LE2 did not (Fig. 5A). In contrast, E2 and LE2 increased mRNA expression of genes encoding components of the alternative pathway (Fig. 5B–D). In the case of Cfb, DPN showed a character different from E2, i.e. restored mRNA expression (Fig. 5B).

We examined mRNA expression of Tnfa, Il1b, Il6 and Il12b, which showed no regulation by ovarian hormones (data not shown). We also tested expression of anti-inflammatory cytokines Il4 and Il10, but their mRNA levels were below the detection limit of TaqMan-based real-time PCR.

### Ovarian Hormone-dependent Regulation of Aromatase and Estrogen Receptor Genes

**Figure 6:**
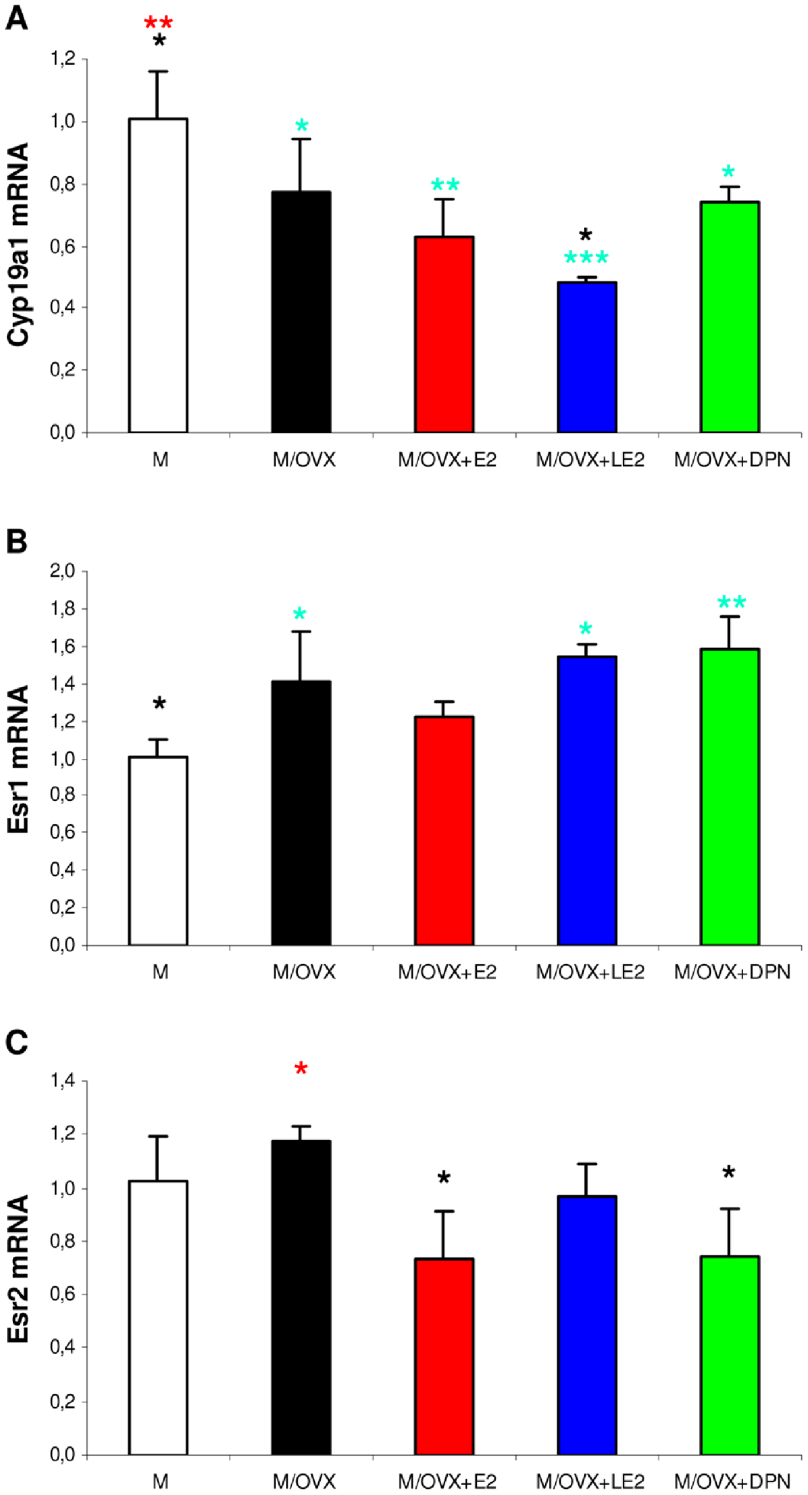
The impact of gonadectomy and ER agonist treatments on mRNA expression of aromatase and ER genes in the hippocampus of middle-aged OVX rats. Expression of Cyp19a1 (A), Esr1 (B) and Esr2 (C) was measured by real-time PCR. ANOVA revealed statistically significant treatment effects for each gene (p<0.001 for Cyp19a1, p = 0.006 for Esr1 and p = 0.028 for Esr2). Turquoise, black and red asterisks indicate statistically significant group differences compared to M, M/OVX and M/OVX+E2 animals, respectively, by Newman-Keuls post hoc test. * corresponds to 0.01<P<0.05, ** to 0.001<P<0.01 and *** to P<0.001.

In the absence of circulating E2, local synthesis determines the actual E2 concentration in the hippocampus of OVX rats. In this process, cytochrome P450 aromatase (encoded by Cyp19a1) carries out hydroxylation of the A ring of androgen precursors, androstendione and testosterone [56]. Cyp19a1 expression showed 1.4-fold (p = 0.025) decrease in middle-aged OVX rats compared to controls (Fig. 6A). E2 and isotype-selective ER agonists did not restore mRNA expression of Cyp19a1, LE2 even decreased it further (Fig. 6A). It is known that aromatase activity has been involved in many functions, from synaptogenesis to neuroprotection [57]. In accord with decreased Cyp19a1, we found downregulation of growth associated protein 43 (RQ = 0.827, p = 0.018) and synaptophysin (RQ = 0.876, p = 0.022). We examined the correlation between the expression of Gap43, Cyp19a1 and macrophage-associated genes. In M/OVX animals, Pearson’s correlation analysis revealed positive correlation between Gap43 and Cyp19a1 (R = 0.73, p = 0.162), and negative correlation between Gap43 and Cd11b (R = −0.92, p = 0.077), Cyp19a1 and Cd11b (R = −0.81, p = 0.094), Cyp19a1 and Cd22 (R = −0.88, p = 0.052). These results suggested that a positive correlation might exist between the expression of Gap43 and Cyp19a1 in the hippocampus of middle-aged OVX rats. In addition, strong negative correlation might exist between the expression of Gap43 and Cd11b, Cyp19a1 and Cd22.

Among ER genes, Esr1, encoding ERα, was upregulated 1.4-fold (p = 0.033) in OVX rats compared to controls (Fig. 5B). The expression of Esr2 (Fig. 5C) and Gper encoding ERβ and GPR30, respectively, did not change after ovariectomy. E2 attenuated mRNA expression of Esr1, while isotype-selective ER agonists did not (Fig. 5B). E2 and DPN significantly reduced expression of Esr2 compared to M/OVX animals (Fig. 5C).

### Data Analysis Reveals Upregulation of Macrophage-associated Genes and Complement in Postmenopausal Women

In order to address the effect of menopause on the innate immune system in the hippocampus, we compared the expression of selected genes in raw microarray data of pre- and postmenopausal women [6]. We identified a large series of changes related to menopause (and aging) that were strikingly similar to those induced by ovariectomy in middle-aged rats. These included upregulation of CD22 (FC = 2.4), CD45 (FC = 2.3), CD11b (FC = 1.5), CD18 (FC = 5.1), IBA1 (FC = 1.7), CD14 (FC = 4.1), C3 (FC = 2.1), and down-regulation of GAP43 (FC = 0.55), SYP (FC = 0.61), SEMA3A (FC = 0.756). Additional similarities included unaltered expression of astroglia markers and proinflammatory cytokines.

## Discussion

Focusing on the innate immune system in the hippocampus, we studied the impact of ovarian hormone deficiency and subsequent estrogen treatments with E2 and isotype-selective ER agonists on gene expression in middle-aged female rats. From the results we conclude that ovarian hormone deficiency alerts the innate immune system in the hippocampus, and subsequent E2 replacement is capable of counteracting several changes via ERα and ERβ. Our findings are in line with previous reports that have demonstrated relationship between endogenous estrogen status and microglia reactivity [20], escape of microglia from inhibitory neuronal control [58], activation of microglia [34], [35], [59] and enhanced complement expression in the normal aged brain [6], [8], [60].

### Ovariectomy Alerts the Innate Immune System in the Hippocampus

#### Ovariectomy sensitizes microglia

Microglia show plasticity and possess various priming states that determine the microglial response to subsequent stimuli. Most studied examples include the classical (M1) and alternative (M2) priming states that evolve in response to interferon-γ and IL4 (or IL13), respectively. In response to ovarian hormone deficiency, we find elevated expression of several microglia markers (Iba1, Cd68, Cd80), phagocytic receptors (Cd11b, Cd18, Fcgr1a, Fcgr2b) and recognition molecules (Tlr3, Tlr9) in the hippocampus. Despite elevated expression of macrophage-associated genes, glial cells do not produce proinflammatory cytokines including tumor necrosis factor α (TNFα). This is in accord with the finding of a recent study demonstrating that while age-dependent microglial NF-κB activation and TNFα overproduction take place in the hypothalamus, only minor NF-κB activation occurs in the cerebral cortex of middle-aged mice [61]. On the other hand, several studies have reported elevated cytokine production in aging microglia isolated from rats and mice. Enhanced production of cytokines in aged microglia may represent age-related alterations. The apparent disagreement between the findings in aged and middle-aged animals suggests that in middle-aged female rats, elevation of cytokine production has not started yet.

The alterations in hippocampal gene expression suggest sensitization of microglia, characterized by moderate upregulation of Iba1, Cd68, Cd11b, Cd18, Fcgr1a, Fcgr2b and Cd80. Of note, mRNA expression of Cd74, Cd86, Cd163, Mrc1, Fcgr2b, Cd14, Tlr4 does not change after ovariectomy. Analysis of human microarray data and comparison between pre- and postmenopausal individuals reveal analogous changes in the expression of these genes indicating that microglia sensitization may occur in postmenopausal women. This special phenotype suggests that microglia adapt and maintain homeostasis by increasing the expression of phagocytic receptors and co-receptors to possess enhanced phagocytic potential.

#### Ovariectomy alters the neuronal control of microglia reactivity

CD22, formerly known as B-lymphocyte cell adhesion molecule, is expressed in rodent neurons [62]. Neurons secrete a soluble form of CD22, which inhibits microglial activation and proinflammatory cytokine production *in vitro* via CD45 [62]. CD45 is the most abundantly expressed transmembrane protein-tyrosine phosphatase. Quiescent microglial cells constitutively express CD45, which elevates further during activation [63]. We find weak mRNA expression of Cd22 and Cd45 in the hippocampus of middle-aged female rats, both of which increase after ovariectomy. In agreement with elevated expression of Cd22 and Cd45, mRNA expression of proinflammatory cytokines does not change significantly either. Interestingly, these alterations are reminiscent to those we observed in the hippocampus of postmenopausal women. These results suggest that in OVX rats and postmenopausal women neurons enhance inhibitory CD22-CD45 signaling which prevents the transition from surveying to effector microglia.

Ovariectomy induces a moderate increase in Cd200r1 expression in middle-aged rats. This finding suggests that in the hippocampus of ovarian hormone deprived animals microglial cells enhance the expression of receptors for inhibitory ligands including Cd45 and Cd200r1. On the other hand, mRNA expression of Cx3cr1, Nrp1 and Sirpa does not change in middle-aged ovariectomized rats.

Another characteristic change which may contribute to the alteration of neuron-microglia communication is the decrease of Sema3a expression. Of note, downregulation of SEMA3A takes place in postmenopausal woman as well. Neurons secrete Sema3A, which can navigate axons, regulate neuronal polarization [64] and facilitate axonal transport [65]. Stressed neurons also secrete Sema3A, which promotes microglial production of growth factors and anti-inflammatory cytokines *in vitro* [66]. Sema3A also induces apoptosis of activated microglia [67] indicating that stressed neurons can protect themselves from activated microglia. Therefore, decreased expression of Sema3a may modulate both neuronal and glial functions via the aforementioned mechanisms.

#### Ovariectomy upregulates C3, factor B and properdin

Complement is a powerful proteolytic cascade which participates in the elimination of invading pathogens, apoptotic cells, and in the regulation of the innate immune response and inflammatory reactions [68]. Age-dependent upregulation of C1q and C3 has been described in the rodent and human brain [6], [8], [60]. We show ovarian hormone-dependent upregulation of C3 in the hippocampus of middle-aged ovariectomized rats and postmenopausal women. In middle-aged rats, we find low level expression of components of the alternative pathway, and observe upregulation of Cfb and properdin after ovariectomy. Increased expression of alternative pathway components can result in elevated tick-over [69], properdin-dependent [70] and amplification mechanisms [71]. However, expression of complement regulators such as Crry and Cd55 does not change. It has potential functional significance as activation of the alternative pathway may be connected to the initial activation of microglial cells. In mice, deletion of Crry induces microglial priming, but inactivation of the alternative pathway in Crry−/− suspends the shift in the phenotype suggesting that the alternative pathway may play a role in microglia activation [72]. Apparent coincidence in the alterations of macrophage-associated and complement gene expression tempts us to speculate that elevated expression of C3, Cfb and Cfp may contribute to the initial activation of microglial cells after the cessation of ovarian hormone synthesis.

#### Ovariectomy alters expression of cytochrome P450 aromatase and ERα

Local estrogen synthesis in the hippocampus provides high E2 concentration [56], [73]. We show that expression of Cyp19a1 aromatase decreases in the absence of circulating ovarian hormones. Earlier studies have demonstrated that expression of synaptic proteins is regulated by aromatase activity in the hippocampus [56]. In concert with decreased Cyp19 expression, we find down-regulation of synaptic marker genes including Gap43 and Syp. These findings suggest that local E2 level may decrease after the loss of circulating E2 and progesterone from ovarian sources. We reveal that in middle-aged rats ovariectomy increases ERα expression, but has no effect on ERβ and GPR30 in the hippocampus. It is important to note that these results reflect the changes of gene expression from a wide range of cell types including neurons, astrocytes and microglia. It is known that at hippocampal synapses ERα is predominantly concentrated presynaptically and highly enriched with synaptic vesicles [74]. Age decreases ERα level and/or localization in the hippocampus [11]. Taking together, following ovariectomy hippocampal neurons and glia respond to the sudden drop of serum levels of ovarian hormones by downregulation of aromatase, upregulation of macrophage-associated genes and receptors for inhibitory neuronal ligands. Altered expression of inhibitory neuronal ligands may contribute to maintain the tight control of microglia reactivity in middle-aged female rats.

### E2 Replacement Modulates the Expression of Macrophage-associated Genes via ERα and ERβ

Ovariectomy induces a mild change in the phenotype of microglial cells. E2 replacement restores the alterations in the case of Iba1, Cd68, Cd11b and Cd18. These genes are likely to be regulated only by E2, i.e. progesterone does not modulate them. Both ERα and ERβ agonist treatments mitigate mRNA expression of these genes. Of note, E2 replacement does not reverse ovariectomy-evoked upregulation of Fcgr2b and Tlr3. These genes are likely to be regulated by E2 and progesterone. In addition, E2 and ERα agonist LE2 treatments trigger a marked increase in mRNA expression of Mrc1 and Cd163, which are widely used as M2 activation markers [75], [76], indicating that E2 may differently regulates Mrc1 and Cd163 in the presence or in the absence of progesterone. These findings are in agreement with previous data demonstrating the role of ERα in the estrogenic regulation of microglia [19], [28], [30]. On the other hand, inhibition of Iba1, Cd68, Cd11b and Cd18 mRNA expression by ERβ agonist DPN may support the view that microglia also express ERβ [29], [31]. However, we can not exclude the possibility that the effect of DPN is an indirect effect, i.e. the result of the interaction of microglia with other cell types.

Neuronal control of microglia reactivity has also significant impact on the innate immune system of the brain [77]. Of note, E2 and isotype-selective ER agonists enhance the expression of microglial receptors for inhibitory neuronal ligands including Cd45, Cd200r1 and Nrp1. The E2-like effects of DPN on the expression of Cd45 and Nrp1 can support the notion that microglial cells express low level of ERβ. However, different effects of E2 and DPN on Cd200r expression do not confirm the presence of ERβ in microglia. Estrogens affect the expression of inhibitory neuronal ligands as well. E2 replacement reverses Sema3a expression primarily via ERα, although ERβ agonist DPN is also effective. Neither E2 nor isotype-selective ER agonists turn the elevation of Cd22 expression in OVX rats.

Summing up, our findings indicate that the innate immune system of the hippocampus in middle-aged rats is responsive to the gonadal hormone milieu. Ovariectomy triggers sensitization of microglia, elevation of complement expression especially components of the alternative pathway, and decline of aromatase level. Although E2 replacement does not influence mRNA expression of aromatase, E2 decreases the expression of microglia markers and phagocytic receptors in part, and increases M2 activation markers indicating a shift towards a protective microglia phenotype. E2 also modulates complement expression, attenuates C3 and enhances Cfb, Cfh expression. In addition, E2 partly restores Sema3A. The ERβ agonist DPN attenuates both microglia sensitization and complement expression. Our results suggest that the innate immune system is responsive to estrogen replacement, at least for a short period of time after ovariectomy.

## References

[1] Buzsáki G, Moser EI (2013) Memory, navigation and theta rhythm in the hippocampal-entorhinal system. Nat Neurosci 16: 130–138. 23354386 10.1038/nn.3304 PMC4079500

[2] Blalock EM, Chen KC, Sharrow K, Herman JP, Porter NM, et al (2003) Gene microarrays in hippocampal aging: statistical profiling identifies novel processes correlated with cognitive impairment. J Neurosci 23: 3807–3819. 12736351 10.1523/JNEUROSCI.23-09-03807.2003 PMC6742177

[3] Verret L, Mann EO, Hang GB, Barth AM, Cobos I, et al (2012) Inhibitory interneuron deficit links altered network activity and cognitive dysfunction in Alzheimer model. Cell 149: 708–721. 22541439 10.1016/j.cell.2012.02.046 PMC3375906

[4] Kuhn HG, Dickinson-Anson H, Gage FH (1996) Neurogenesis in the dentate gyrus of the adult rat: age-related decrease of neuronal progenitor proliferation. J Neurosci 16: 2027–2033. 8604047 10.1523/JNEUROSCI.16-06-02027.1996 PMC6578509

[5] Bakker A, Krauss GL, Albert MS, Speck CL, Jones LR, et al (2012) Reduction of hippocampal hyperactivity improves cognition in amnestic mild cognitive impairment. Neuron 74: 467–474. 22578498 10.1016/j.neuron.2012.03.023 PMC3351697

[6] Berchtold NC, Cribbs DH, Coleman PD, Rogers J, Head E, et al (2008) Gene expression changes in the course of normal brain aging are sexually dimorphic. Proc Natl Acad Sci, USA 105: 15605–15610. 18832152 10.1073/pnas.0806883105 PMC2563070

[7] McKinlay S, Brambilla D, Posner J (1992) The normal menopausal transition. Maturitas 14: 103–115. 1565019 10.1016/0378-5122(92)90003-m

[8] Kadish I, Thibault O, Blalock EM, Chen KC, Gant JC, et al (2009) Hippocampal and cognitive aging across the lifespan: a bioenergetic shift precedes and increased cholesterol trafficking parallels memory impairment. J Neurosci 29: 1805–1816. 19211887 10.1523/JNEUROSCI.4599-08.2009 PMC2661568

[9] Loy R, Gerlach JL, McEwen BS (1988) Autoradiographic localization of estradiol-binding neurons in the rat hippocampal formation and entorhinal cortex. Brain Res 467: 245–251. 3378173 10.1016/0165-3806(88)90028-4

[10] Shughrue PJ, Merchenthaler I (2000) Evidence for novel estrogen binding sites in the rat hippocampus. Neurosci 99: 605–612. 10.1016/s0306-4522(00)00242-6 10974424

[11] Adams MM, Fink SE, Shah RA, Janssen WG, Hayashi S, et al (2002) Estrogen and aging affect the subcellular distribution of estrogen receptor-alpha in the hippocampus of female rats. J Neurosci 22: 3608–3612. 11978836 10.1523/JNEUROSCI.22-09-03608.2002 PMC6758372

[12] Waters EM, Torres-Reveron A, McEwen BS, Milner TA (2008) Ultrastructural localization of extranuclear progestin receptors in the rat hippocampal formation. J Comp Neurol 511: 34–46. 18720413 10.1002/cne.21826 PMC2577145

[13] Aenlle KK, Kumar A, Cui L, Jackson TC, Foster TC (2009) Estrogen effects on cognition and hippocampal transcription. Neurobiol Aging 30: 932–945. 17950954 10.1016/j.neurobiolaging.2007.09.004 PMC2730158

[14] Zhao L, Morgan TE, Mao Z, Lin S, Cadenas E, et al (2012) Continuous versus cyclic progesterone exposure differentially regulates hippocampal gene expression and functional profiles. PLoS One 7: e31267. 22393359 10.1371/journal.pone.0031267 PMC3290616

[15] Dominguez R, Hu E, Zhou M, Baudry M (2009) 17beta-estradiol-mediated neuroprotection and ERK activation require a pertussis toxin-sensitive mechanism involving GRK2 and beta-arrestin-1. J Neurosci 29: 4228–4238. 19339617 10.1523/JNEUROSCI.0550-09.2009 PMC3182118

[16] Witty CF, Foster TC, Semple-Rowland SL, Daniel JM (2012) Increasing hippocampal estrogen receptor alpha levels via viral vectors increases MAP kinase activation and enhances memory in aging rats in the absence of ovarian estrogens. PLoS One 7: e51385. 23240018 10.1371/journal.pone.0051385 PMC3519866

[17] McEwen BS, Akama KT, Milner TA, Spencer-Segal JL, Waters EM (2012) Estrogen effects on the brain: actions beyond the hypothalamus via novel mechanisms. Behav Neurosci 126: 4–16. 22289042 10.1037/a0026708 PMC3480182

[18] Wu WW, Bryant DN, Dorsa DM, Adelman JP, Maylie J (2013) Ovarian hormone loss impairs excitatory synaptic transmission at hippocampal CA3-CA1 synapses. J Neurosci 33: 16158–16169. 24107948 10.1523/JNEUROSCI.2001-13.2013 PMC3792457

[19] Vegeto E, Belcredito S, Etteri S, Ghisletti S, Brusadelli A, et al (2003) Estrogen receptor-α mediates the brain anti-inflammatory activity of estradiol. Proc Natl Acad Sci, USA 100: 9614–9619. 12878732 10.1073/pnas.1531957100 PMC170966

[20] Vegeto E, Belcredito S, Ghisletti S, Meda C, Etteri S, et al (2006) The endogenous estrogen status regulates microglia reactivity in animal models of neuroinflammation. Endocrinol 147: 2263–2272. 10.1210/en.2005-1330 16469811

[21] Tiwari-Woodruff S, Morales LBJ, Lee R, Voskuhl RR (2007) Differential neuroprotective and antiinflammatory effects of estrogen receptor (ER) α and ERβ ligand treatment. Proc Natl Acad Sci, USA 104: 14813–14818. 17785421 10.1073/pnas.0703783104 PMC1976208

[22] Lewis DK, Johnson AB, Stohlgren S, Harms A, Sohrabji F (2008) Effects of estrogen receptor agonists on the regulation of the inflammatory response in astrocytes from young adult and middle-aged female rats. J Neuroimmunol 195: 47–59. 18328572 10.1016/j.jneuroim.2008.01.006 PMC2394738

[23] Loram LC, Sholar PW, Taylor FR, Wiesler JL, Babb JA, et al (2012) Sex and estradiol influence glial proinflammatory responses to lipopolysaccharide in rats. Psychoneuroendocrinol 37: 1688–1699. 10.1016/j.psyneuen.2012.02.018 PMC3417083 22497984

[24] Weber MT, Rubin LH, Maki PM (2013) Cognition in perimenopause: the effect of transition stage. Menopause 20: 511–517. 23615642 10.1097/GME.0b013e31827655e5 PMC3620712

[25] Epperson CN, Sammel MD, Freeman EW (2013) Menopause effects on verbal memory: findings from a longitudinal community cohort. J Clin Endocrinol Metab 98: 3829–3838. 23836935 10.1210/jc.2013-1808 PMC3763981

[26] Ji K, Akgul G, Wollmuth LP, Tsirka SE (2013) Microglia actively regulate the number of functional synapses. PLoS One 8: e56293. 23393609 10.1371/journal.pone.0056293 PMC3564799

[27] Schafer DP, Lehrman EK, Kautzman AG, Koyama R, Mardinly AR, et al (2012) Microglia sculpt postnatal neural circuits in an activity and complement-dependent manner. Neuron 74: 691–705. 22632727 10.1016/j.neuron.2012.03.026 PMC3528177

[28] Sierra A, Gottfried-Blackmore AC, Milner T, McEwen BS, Bulloch K (2008) Steroid hormone receptor expression and function in microglia. Glia 56: 659–674. 18286612 10.1002/glia.20644

[29] Saijo K, Collier JG, Li AC, Katzenellenbogen JA, Glass CK (2011) An ADIOL-ERβ-CtBP transrepression pathway negatively regulates microglia-mediated inflammation. Cell 145: 584–595. 21565615 10.1016/j.cell.2011.03.050 PMC3433492

[30] Crain JM, Nikodemova M, Watters JJ (2013) Microglia express distinct M1 and M2 phenotypec markers in the postnatal and adult central nervous system in male and female mice. J Neurosci Res 91: 1143–1151. 23686747 10.1002/jnr.23242 PMC3715560

[31] Wu WF, Tan XJ, Dai YB, Krishnan V, Warner M, et al (2013) Targeting estrogen receptor β in microglia and T cells to treat experimental autoimmune encephalomyelitis. Proc Natl Acad Sci, USA 110: 3543–3548. 23401502 10.1073/pnas.1300313110 PMC3587193

[32] Gibson C, Constantin D, Prior M, Bath P, Murphy S (2005) Progesterone suppresses the inflammatory response and nitric oxide synthase-2 expression following cerebral ischemia. Exp Neurol 193: 522–530. 15869954 10.1016/j.expneurol.2005.01.009

[33] Hanisch U, Kettenmann H (2007) Microglia: active sensor and versatile effector cells in the normal and pathologic brain. Nat Neurosci 10: 1387–1394. 17965659 10.1038/nn1997

[34] Perry VH, Matyszak MK, Fearn S (1993) Altered antigen expression of microglia in the aged rodent CNS. Glia 7: 60–67. 8423063 10.1002/glia.440070111

[35] Frank M, Barrientos R, Biedenkapp J, Rudy J, Watkins L, et al (2006) mRNA up-regulation of MHC II and pivotal proinflammatory genes in normal brain aging. Neurobiol Aging 27: 717–722. 15890435 10.1016/j.neurobiolaging.2005.03.013

[36] Lynch AM, Murphy KJ, Deighan BF, O’Reilly JA, Gun’ko YK, et al (2010) The impact of glial activation in the aging brain. Aging Dis 1: 262–278. 22396865 PMC3295033

[37] Wong AM, Patel NV, Patel NK, Wei M, Morgan TE, et al (2005) Macrosialin increases during normal brain aging are attenuated by caloric restriction. Neurosci Lett 390: 76–80. 16157452 10.1016/j.neulet.2005.07.058

[38] Downer EJ, Cowley TR, Lyons A, Mills KH, Berezin V, et al (2010) A novel anti-inflammatory role of NCAM-derived mimetic peptide, FGL. Neurobiol Aging 31: 118–128. 18468731 10.1016/j.neurobiolaging.2008.03.017

[39] Daniel JM, Hulst JL, Berbling JL (2006) Estradiol replacement enhances working memory in middle-aged rats when initiated immediately after ovariectomy but not after a long-term period of ovarian hormone deprivation. Endocrinol 147: 607–614. 10.1210/en.2005-0998 16239296

[40] Engler-Chiurazzi E, Tsang C, Nonnenmacher S, Liang WS, Corneveaux JJ, et al (2011) Tonic Premarin dose-dependently enhances memory, affects neurotrophin protein levels and alters gene expression in middle-aged rats. Neurobiol Aging 32: 680–697. 19883953 10.1016/j.neurobiolaging.2009.09.005 PMC3016463

[41] Barha CK, Galea LA (2011) Motherhood alters the cellular response to estrogens in the hippocampus later in life. Neurobiol Aging 32: 2091–2095. 20034703 10.1016/j.neurobiolaging.2009.12.004

[42] Sárvári M, Kalló I, Hrabovszky E, Solymosi N, Tóth K, et al (2010) Estradiol replacement alters expression of genes related to neurotransmission and immune surveillance in the frontal cortex of middle-aged, ovariectomized rats. Endocrinol 151: 3847–3862. 10.1210/en.2010-0375 20534718

[43] Sárvári M, Kalló I, Hrabovszky E, Solymosi N, Tóth K, et al (2011) Estrogens regulate neuroinflammatory genes via estrogen receptors alpha and beta in the frontal cortex of middle-aged female rats. J Neuroinflamm 8: 82. 10.1186/1742-2094-8-82 PMC3161870 21774811

[44] Vandesompele J, De Preter K, Pattyn F, Peppe B, Van Roy N, et al (2002) Accurate normalization of real-time quantitative RT-PCR data by geometric averaging of multiple internal control genes. Genome Biol 3: RESEARCH0034.1–0034.11. 12184808 10.1186/gb-2002-3-7-research0034 PMC126239

[45] Bustin SA, Benes V, Garson JA, Hellemans J, Huggett J, et al (2009) The MIQE guidelines: minimum information for publication of quantitative real-time PCR experiments. Clin Chem 55: 611–622. 19246619 10.1373/clinchem.2008.112797

[46] MAQC Consortium (2006) The microarray quality control (MAQC) project shows inter- and intraplatform reproducibility of gene expression measurements. Nat Biotech 24: 1151–1161. 10.1038/nbt1239 PMC3272078 16964229

[47] Wu Z, Irizarry R, Gentleman R, Martinez-Murillo F, Spencer F (2004) A model-based background adjustment for oligonucleotide expression arrays. J Am Stat Assoc 99: 909–917.

[48] Smyth G (2004) Linear models and empirical Bayes methods for assessing differential expression in microarray experiments. Stat Appl Genet Mol Biol 3: 3. 10.2202/1544-6115.1027 16646809

[49] Benjamini Y, Hochberg Y (1995) Controlling the false discovery rate: a practical and powerful approach to multiple testing. J Royal Stat Soc Series B 57: 289–300.

[50] Gentleman R, Carey V, Bates D, Bolstad B, Dettling M, et al (2004) Bioconductor: Open software development for computational biology and bioinformatics. Genome Biol 5: R80. 15461798 10.1186/gb-2004-5-10-r80 PMC545600

[51] Bowman CC, Rasley A, Tranguch SL, Marriott I (2003) Cultured astrocytes express toll-like receptors for bacterial products. Glia 43: 281–291. 12898707 10.1002/glia.10256

[52] Hillisch A, Peters O, Kosemund D, Müller G, Walter A, et al (2004) Dissecting physiological roles of estrogen receptor α and β with potent selective ligands from structure-based design. Mol Endorinol 18: 1599–1609. 10.1210/me.2004-0050 15105439

[53] Sun J, Baudry J, Katzenellenbogen JA, Katzenellenbogen BS (2003) Molecular basis for the subtype discrimination of the estrogen receptor-beta-selective ligand, diarylpropio-nitrile. Mol Endocrinol 17: 247–258. 12554752 10.1210/me.2002-0341

[54] Veerhuis R, Nielsen HM, Tenner AJ (2011) Complement in the brain. Mol Immunol 48: 1592–1603. 21546088 10.1016/j.molimm.2011.04.003 PMC3142281

[55] Götze O, Müller-Eberhard HJ (1971) The C3-activator system: an alternate pathway of complement activation. J Exp Med 134: 90–108. 19867385 PMC2139059

[56] Prange-Kiel J, Schmutterer T, Fester L, Zhou L, Imholz P, et al (2013) Endocrine regulation of estrogen synthesis in the hippocampus. Prog Histochem Cytochem 48: 49–64. 23906992 10.1016/j.proghi.2013.07.002

[57] Kretz O, Fester L, Wehrenberg U, Zhou L, Brauckmann S, et al (2004) Hippocampal synapses depend on hippocampal estrogen synthesis. J Neurosci 24: 5913–5921. 15229239 10.1523/JNEUROSCI.5186-03.2004 PMC6729232

[58] Jurgens HA, Johnson RW (2012) Dysregulated neuronal-microglial cross-talk during aging, stress and inflammation. Exp Neurol 233: 40–48. 21110971 10.1016/j.expneurol.2010.11.014 PMC3071456

[59] Sheng JG, Mrak RE, Griffin WS (1998) Enlarged and phagocytic, but not primed, interleukin-1 alpha-immunoreactive microglia increase with age in normal human brain. Acta Neuropathol 95: 229–234. 9542587 10.1007/s004010050792

[60] Reichwald J, Danner S, Wiederhold KH, Staufenbiel M (2009) Expression of complement system components during aging and amyloid deposition in APP transgenic mice. J Neuroinflamm 6: 35. 10.1186/1742-2094-6-35 PMC2784442 19917141

[61] Zhang G, Li J, Purkayastha S, Tang Y, Zhang H, et al (2013) Hypothalamic programming of systemic ageing involving IKK-β, NF-κB and GnRH. Nature 497: 211–216. 23636330 10.1038/nature12143 PMC3756938

[62] Mott RT, Ait-Ghezala G, Town T, Mori T, Tan J (2004) Neuronal expression of CD22: novel mechanism for inhibiting microglial proinflammatory cytokine production. Glia 46: 363–379. 10.1002/glia.20009 15095367

[63] Sedgewick JD, Schwender S, Imrich H, Dörries R, Butcher GW, et al (1991) Isolation and direct characterization of resident microglial cells from the normal and the inflamed central nervous system. Proc Natl Acad Sci, USA 88: 7438–74442. 1651506 10.1073/pnas.88.16.7438 PMC52311

[64] Shelley M, Cancedda L, Lim BK, Popescu A, Cheng P, et al (2011) Semaphorin3A regulates neuronal polarization by suppressing axon formation and promoting dendrite growth. Neuron 71: 433–446. 21835341 10.1016/j.neuron.2011.06.041 PMC3164872

[65] Yamane M, Yamashita N, Yamamoto H, Iizuka A, Shouji M, et al (2012) Semaphorin3A facilitates axonal transport through a local calcium signaling and tetrodotoxin-sensitive voltage-gated sodium channels. Biochem Biophys Res Com 422: 333–338. 22575508 10.1016/j.bbrc.2012.05.003

[66] Minghetti L, Ajmone-Cat MA, De Berardinis MA, De Simone R (2005) Microglial activation in chronic neurodegenerative diseases: roles of apoptotic neurons and chronic stimulation. Brain Res Rev 48: 251–256. 15850664 10.1016/j.brainresrev.2004.12.015

[67] Majed HH, Chandran S, Niclou SP, Nicholas RS, Wilkins A, et al (2006) A novel role for Sema3A in neuroprotection from injury mediated by activated microglia. J Neurosci 26: 1730–1738. 16467521 10.1523/JNEUROSCI.0702-05.2006 PMC6793642

[68] Ricklin D, Hajishengallis G, Yang K, Lambris JD (2010) Complement: a key system for immune surveillance and homeostasis. Nat Immunol 11: 785–797. 20720586 10.1038/ni.1923 PMC2924908

[69] Pangburn MK, Schreiber RD, Müller-Eberhard HJ (1981) Formation of the initial C3 convertase of the alternative complement pathway. Acquisition of C3b-like activities by spontaneous hydrolysis of the putative thioester in native C3. J Exp Med 154: 856–867. 6912277 10.1084/jem.154.3.856 PMC2186450

[70] Fearon DT, Austen KF (1975) Properdin: initiation of alternative complement pathway. Proc Natl Acad Sci, USA 72: 3220–3224. 1059108 10.1073/pnas.72.8.3220 PMC432954

[71] Harboe M, Ulvund G, Vien L, Fung M, Mollnes TE (2004) The quantitative role of alternative pathway amplification in classical pathway induced terminal complement activation. Clin Exp Immunol 138: 439–446. 15544620 10.1111/j.1365-2249.2004.02627.x PMC1809239

[72] Ramaglia V, Hughes T, Donev R, Ruseva M, Wu X, et al (2012) C3-dependent mechanism of microglial priming relevant to multiple sclerosis. Proc Natl Acad Sci, USA 109: 965–970. 22219359 10.1073/pnas.1111924109 PMC3271873

[73] Mukai H, Kimoto T, Hojo Y, Kawato S, Murakami G, et al (2010) Modulation of synaptic plasticity by brain estrogen in the hippocampus. Biochim Biophys Acta 1800: 1030–1044. 19909788 10.1016/j.bbagen.2009.11.002

[74] Tabatadze N, Smejkalova T, Woolley CS (2013) Distribution and posttranslational modification of synaptic ERα in the adult female rat hippocampus. Endocrinol 154: 819–30. 10.1210/en.2012-1870 PMC3548183 23183182

[75] Fenn AM, Henry CJ, Huang Y, Dugan A, Godbout JP (2012) Lipopolysaccharide-induced interleukin (IL)-4 receptor-α expression and corresponding sensitivity to the M2 promoting effects of IL-4 are impaired in microglia of aged mice. Brain Behav Immun 26: 766–777. 22024136 10.1016/j.bbi.2011.10.003 PMC3288757

[76] Vogel DY, Vereyken EJ, Glim JE, Heijnen PD, Moeton M, et al (2013) Macrophages in inflammatory multiple sclerosis lesions have an intermediate activation status. J Neuroinflamm 10: 35. 10.1186/1742-2094-10-35 PMC3610294 23452918

[77] Tian L, Rauvala H, Gahmberg CG (2009) Neuronal regulation of immune responses in the central nervous system. Trends Immunol 30: 91–99. 19144568 10.1016/j.it.2008.11.002

